# Population-specific risk models and AI in clinical practice: Are we ready for the next step in managing common disorders?

**DOI:** 10.1016/j.clinme.2025.100329

**Published:** 2025-05-17

**Authors:** Kartik Kumar, Ponnusamy Saravanan

**Affiliations:** a Department of Respiratory Medicine, Hammersmith Hospital, Imperial College Healthcare NHS Trust, London, UK; b National Heart and Lung Institute, Imperial College London, London, UK; c Warwick Medical School, Warwick Applied Health, University of Warwick, Coventry, UK; d Centre for Global Health, University of Warwick, Coventry, UK; e Diabetes, Endocrinology and Metabolism, George Eliot Hospital NHS Trust, Nuneaton, UK

It was a pleasure to commission the respiratory CME series with Dr Kumar, as it took me back to my days at the front door and memories of my own time in acute take. Dr Kumar’s insight has been invaluable in shaping and finalising the selection of topics, which we hope will be of a great value for practising clinicians. Acute and chronic respiratory presentations continue to remain at the heart of the acute medical take, while pulmonary manifestations of systemic diseases are frequently encountered in a range of medical specialty clinics. In our joint editorial, we offer a concise overview for the readers on the topics covered in this series, through our shared lens.

The sensation of breathlessness may have many causes and can range in severity from mild to debilitating. Unexplained dyspnoea requires consideration of various interacting pathophysiological mechanisms. Guided by the clinical history, advanced testing modalities may be need to considered, including cardiopulmonary exercise testing, ventilation perfusion single photon emission computed tomography with CT, cardiac MRI, bronchoscopy/laryngoscopy and respiratory muscle function testing.^1^ The impact of occupational exposures on respiratory symptoms such as breathlessness and cough must also be considered. Work-related asthma, pneumoconioses, asbestosis, hypersensitivity pneumonitis and chronic obstructive pulmonary disease are among the pulmonary conditions that can be attributable to respiratory hazards in the workplace. A detailed occupational history and early to referral to occupational lung disease specialists are vital.^2^

Careful consideration should be given to whether there may be potentially unifying diagnoses when there are a constellation of seemingly unrelated symptoms. Sarcoidosis, known as the great mimicker, is a multisystem disease characterised by granulomatous inflammation. Initial presentation may be to a range of different medical specialties and it is therefore important to remain vigilant to clues in the clinical history and physical examination findings that may be suggestive of the diagnosis.^3^ Collison *et al* summarise an under-recognised and rare inherited disorder, primary ciliary dyskinesia (PCD), which is characterised by neonatal respiratory distress and chronic sinopulmonary symptoms but can present as subfertility. It highlights the need for recognition and prompt referral for early diagnosis and optimisation of long-term management.^4^

There have been a number of significant developments in the clinical practice of respiratory medicine in recent years. With nearly 1,500 lung transplants performed in the UK between 2014 and 2024, it is crucial for physicians to understand how individuals are selected for lung transplantation and to appreciate pertinent clinical management considerations in postoperative individuals who present acutely to their local healthcare services.^5^ British Thoracic Society guidelines on the management of pleural disease were updated in 2023 and as such this is a timely opportunity to highlight key clinical considerations in the management of pneumothoraces.^6^ Karir *et al*^7^ offer a valuable update on the evolving role of interventional bronchoscopy, highlighting its expanding applications in pulmonary nodules, lung cancer, emphysema, asthma and interstitial lung disease.

Finally, this issue features an excellent collection of original articles from authors around the world. I have selected two contributions as my choices for the issue, both focusing on common cardiovascular disorders.

In the first, Osataphan *et al*^8^ evaluated the ARIC heart failure risk score in a prospective cohort of nearly 9,000 Thai patients and developed a population-specific version – the ARIC-CORE-HF score. Their study demonstrated improved predictive performance and risk stratification, underscoring the importance of population-specific approaches even for common conditions for better management strategies. The second article, by Yang *et al*,^9^ addresses acute myocardial infarction, which remains a leading cause of in-hospital mortality worldwide. While traditional risk factors have long been used for prognosis, the authors employed machine learning techniques on a large, retrospective multicentre dataset of over 12,000 critically ill hospitalised patients across the USA. Their findings represent a step toward integrating artificial intelligence into routine clinical practice – a future that may arrive sooner than we believe.

## Funding

No funding.

## Author contributions

PS commissioned this and wrote the editorial together with KK.

## Declaration of competing interests

KK - Associate editor and editorial board member of *Clinical Medicine* and handled the peer review of the invited CME articles; PS - Commissioned this and wrote the editorial together with KK.
